# Supplementary figures and images for: Patterns of Routes of Administration and Drug Tampering for Nonmedical Opioid Consumption: Data Mining and Content Analysis of Reddit Discussions
Source: J Med Internet Res. 2021 Jan 4;23(1):e21212. doi: 10.2196/21212 (PMC7813634; doi:10.2196/21212)

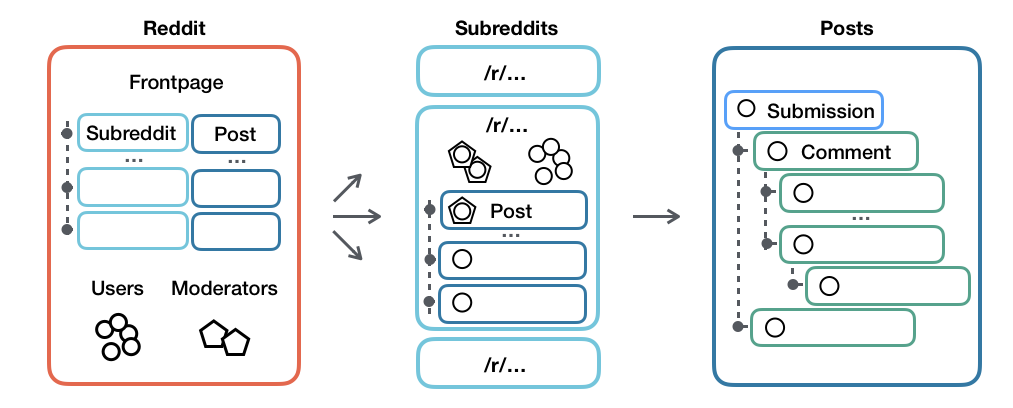

Supplement: Multimedia Appendix 1 [file jmir_v23i1e21212_app1.png]

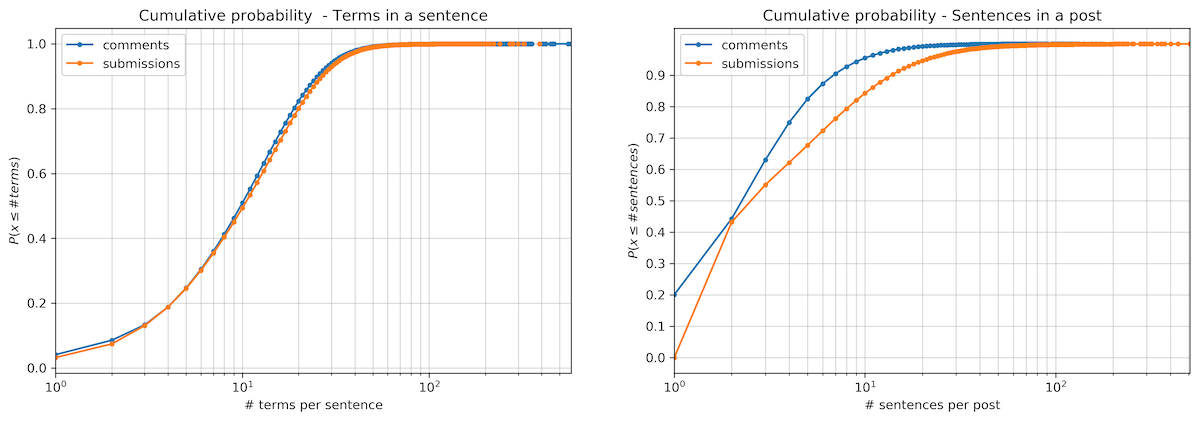

Supplement: Multimedia Appendix 4 [file jmir_v23i1e21212_app4.png]

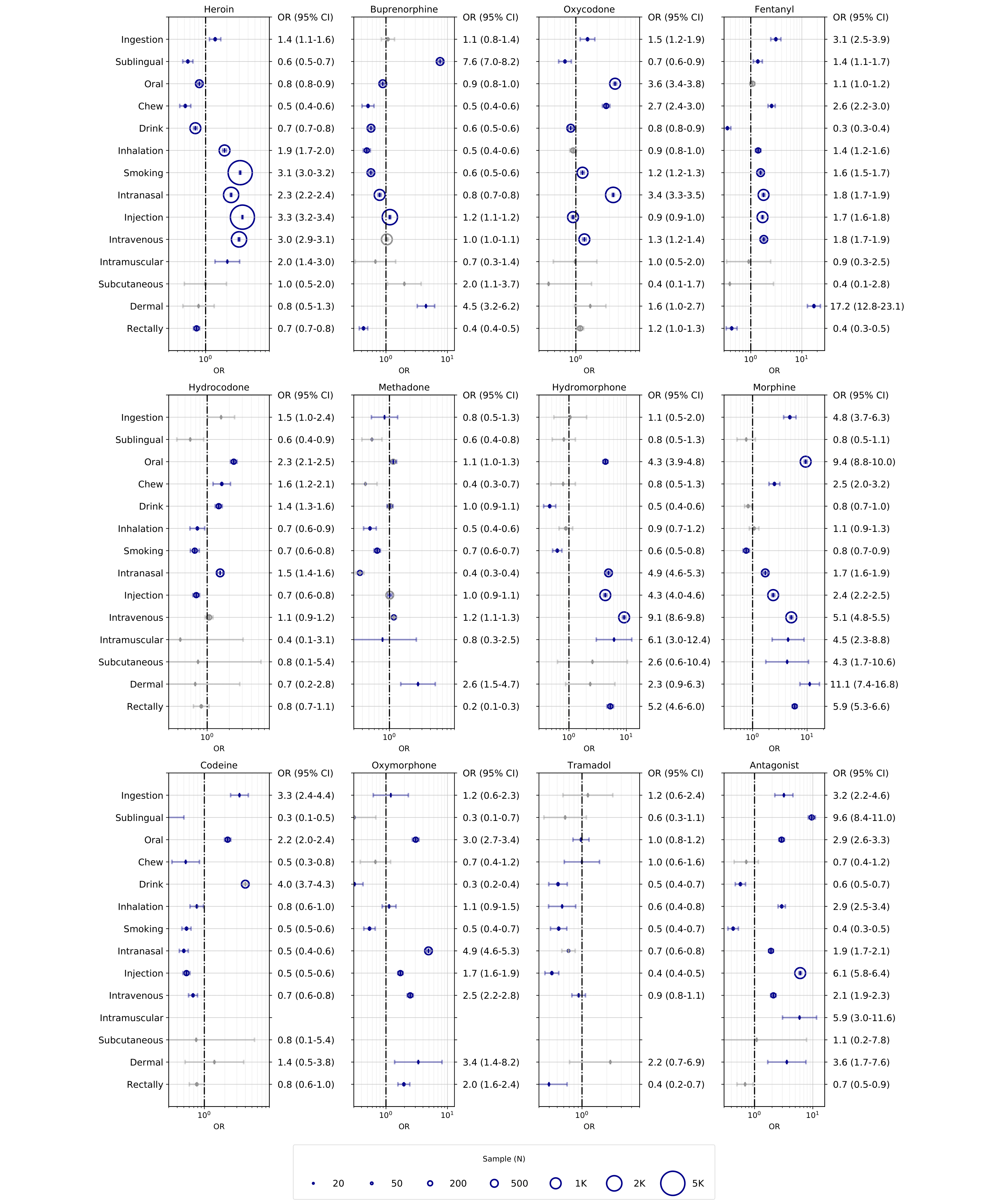

Supplement: Multimedia Appendix 5 [file jmir_v23i1e21212_app5.png]

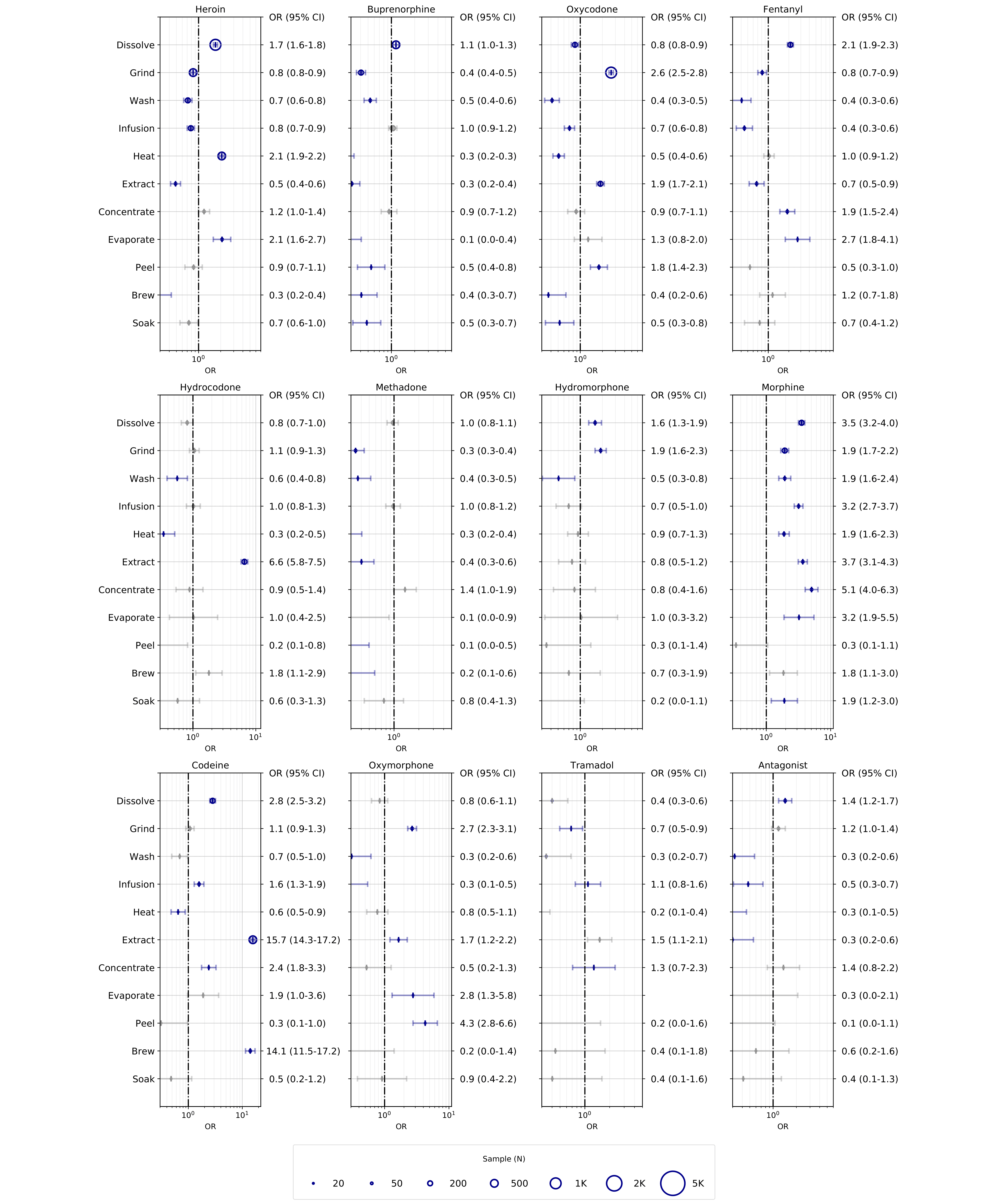

Supplement: Multimedia Appendix 6 [file jmir_v23i1e21212_app6.png]

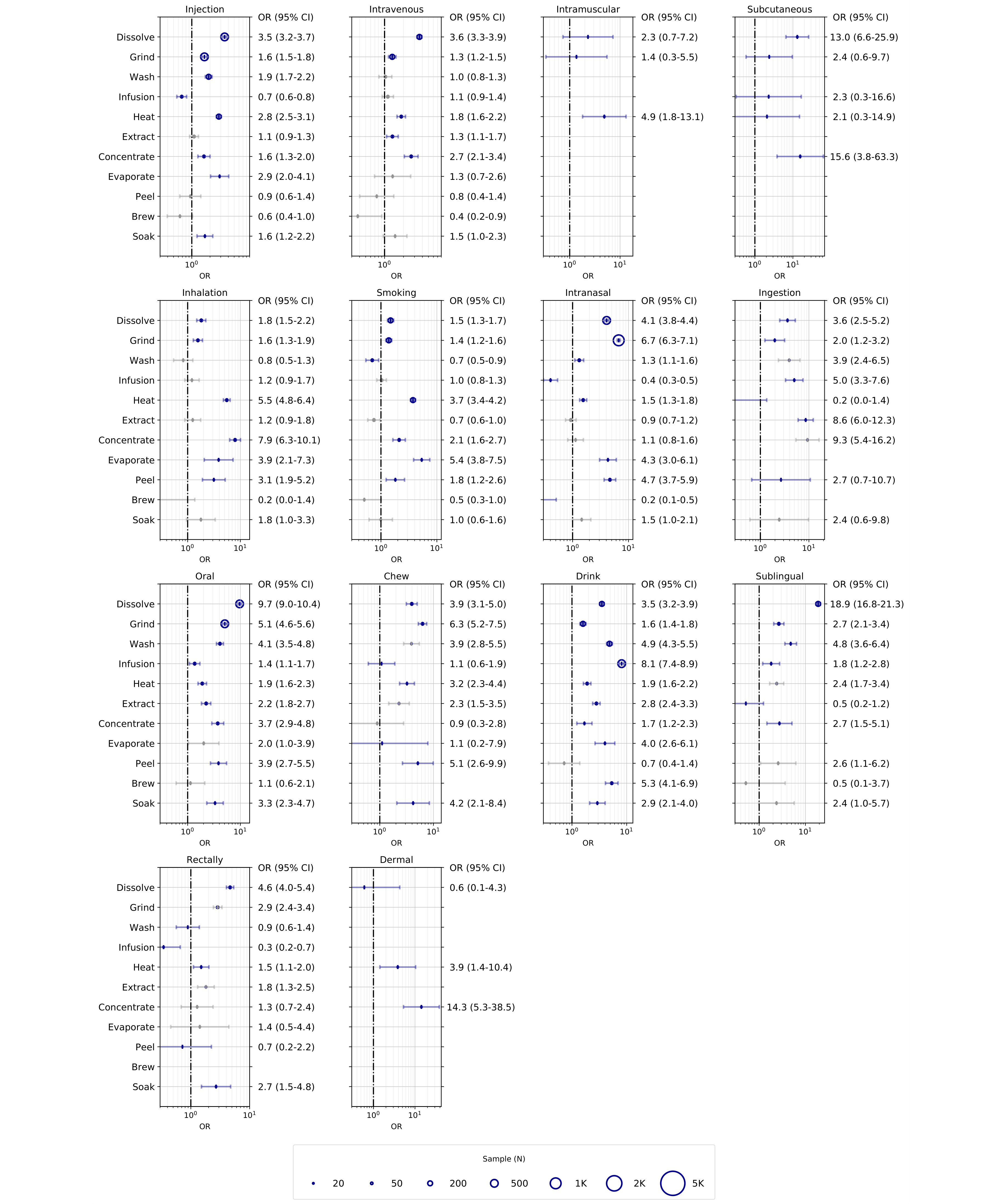

Supplement: Multimedia Appendix 7 [file jmir_v23i1e21212_app7.png]
